# The Potential Systemic Anti-Inflammatory Effect of Turmeric Dried Extract

**DOI:** 10.2174/0118715303329562241116045410

**Published:** 2025-01-08

**Authors:** Concetta Montanino, Federica Farinella, Bruna De Felice, Andrea Del Buono, Armando D’Orta

**Affiliations:** 1 Department of Environmental, Biological and Pharmaceutical Sciences and Technologies, University of Campania Luigi Vanvitelli, Caserta, 81100, Italy;; 2 DDR Research Clinic, Caserta, Italy

**Keywords:** Anti-inflammatory, cholesterol LDL, CRP, ESR, Ferritin, PCR, turmeric

## Abstract

**Background:**

Curcumin is a polyphenolic compound derived from the food spice turmeric that has received interest from the medical and scientific world for its role in the management of several conditions. Clinical studies, in humans, have shown that ingested Curcumin is safe even at high doses (12 g/day), but it has poor bioavailability primarily due to poor absorption and rapid metabolism and elimination. Several strategies have been implemented to improve the bioavailability of Curcumin, for example, the combination of piperine in a complex with Curcumin, or the usage of formulations with phospholipid or liposomal complexes.

**Objective:**

The present work aims to explore and compare the systemic anti-inflammatory effects of two different types of Curcumin: a traditional fat-soluble formulation (95% Curcumin) and an innovative standardized reconstituted water-soluble one (Curcuin), made in micelles in aqueous solution.

**Methods:**

Research was conducted on 30 patients, 15 patients were treated with turmeric (*Curcuma longa L*., rhizome) dried extract titled 95% Curcumin (Curcumin 425mg/day) conjugated with piperine, and 15 patients were treated with Curcumin (turmeric 286 mg dried extract titled 35%; Curcuminoids 100 mg/day, standardized water-soluble) made in micelles in highly absorbed aqueous solution. We considered the quantitative variations of laboratory parameters: Erythrocyte Sedimentation Rate (ESR), C-reactive protein (CRP), Ferritin (24 to 336 ng/mL for adult males), and cholesterol LDL.

**Results and Discussion:**

Patients treated with dried extract titled 95% Curcumin, for 90 days, show a lower value of ESR, CRP, Ferritin, and LDL cholesterol compared with the same laboratory parameters before the introduction of Curcumin into the diet. Also, patients treated with Curcuin report a lower value of ESR, CRP, Ferritin, and LDL cholesterol after the introduction of turmeric dried extract in the diet, but with a major significance compared with those obtained with 95% Curcumin conjugated with piperine.

**Conclusion:**

As we had hypothesized, both turmeric-derived extracts have successfully reduced ESR, CRP, Ferritin, and cholesterol LDL values, exerting an anti-inflammatory action and anti-cholesterolemic action. These results suggest a possible use of Curcumin and in particular Curcuin as a coadjuvant for the treatment of inflammatory disease and to decrease cholesterol levels. However, additional investigation is needed to resolve doubts regarding Curcumin dosage form, dose, and medication frequency.

## 1 INTRODUCTION

Turmeric is a rhizomatous herbaceous permanent plant (*Curcuma longa*) of the ginger family [1, 2]. Curcumin (1,7-bis[4-hydroxy-3-methoxyphenyl]-1,6-heptadiene-3,5-dione), also called diferuloylmethane, is the main natural polyphenol found in the rhizome of *Curcuma longa* (turmeric) and in others Curcuma spp [3, 4]. Most turmeric's pharmacological effects are attributed to natural Curcumin, which is derived from *C. longa* and contains Curcumin I (diferuloylmethane, 94%), Curcumin II (demethoxycurcumin, 6%), and Curcumin III (bisdemethoxycurcumin, 0.3%) [5-7]. Numerous studies have underlined possible antioxidants [8, 9], anti-inflammatories [10, 11], anticancer [12, 13], nephroprotective [14], hypolipidemic [15, 16], hepatoprotective [17], and immunomodulatory [18] characteristics of Curcumin. This suggests that Curcumin, primarily due to its potent anti-inflammatory and immunomodulatory properties, may offer therapeutic benefits for chronic autoimmune conditions such as some forms of rheumatoid arthritis [19, 20]. Additionally, its antioxidant and potential neuroprotective effects make it a promising candidate for slowing the progression of neurodegenerative diseases like Alzheimer's [21-23]. Curcumin modulates various molecular targets, including transcription factors, cytokines, cell cycle proteins, and a multitude of enzymes (COX-II cyclooxygenase), receptors, and cell surface adhesion molecules [24, 25]. Several studies report that a significant effect of Curcuminoid supplementation induces an increase in serum antioxidants’ activities [26]. Curcumin acts as a scavenger against different forms of free radicals, such as ROS and RNS, modulating the activity of GSH, catalase, and SOD enzymes and it can inhibit ROSgenerating enzymes (lipoxygenase/cyclooxygenase and xanthine hydrogenase/oxidase) [27, 28]. In addition, Curcumin, like vitamin E, is considered a chain-breaking antioxidant, scavenging peroxyl radicals [29]. One of the most interesting functions of Curcumin is its anti-inflammatory properties. In fact, at a molecular level, Curcumin seems to block NF-κB activation increased by inflammatory stimuli exerting an anti-inflammatory role [30]; in fact, NF-κB is often overexpressed in chronic disease activated by Tumor Necrosis Factor α (TNF-α) is a major mediator of inflammation, together with pro-inflammatory cytokines [30]. Curcumin is also reported to exercise significant anti-inflammatory and anticancer activities through epigenetic regulation: its effect on DNMT (DNA methyltransferase), enhances the methylation in the promoter region of the oncogenic gene or reduces hypermethylation of cancer’s genes, in dependence of the cancer type [31, 32]. Again, Curcumin has diversified regulatory effects on histone modification including methylation, acetylation, glutathionylation, and phosphorylation and emerging evidence suggests that Curcumin is capable of suppressing cancer by regulation of an array of ncRNAs, especially miRNA, and lncRNAs, which are involved in the growth, migration, invasion, metastasis, and apoptosis [33, 34].

Up to 8–10 g/day in humans, it is a non-toxic and extremely safe chemical, according to several animal and clinical investigations [35]. However, Curcumin's poor bioavailability and poor water solubility significantly limit its therapeutic utility [35, 36]. Many strategies have been set up to improve Curcumin uptake, a common strategy to improve its bioavailability is the use of 95% Curcumin from turmeric-dried extract conjugated to piperine, which has been proven effective in several studies [37, 38]. A novel strategy is represented by Curcuin, by Accuprec Research Labs Pvt, Ltd., is a 95% extract, Curcuma Longa, containing no less than 95% of Curcuminoids (Curcumin, *Demethoxycurcumin* and *Bisdemethoxycurcumin*) with a synergistic combination of sesquiterpenoids (volatile oils) which has been formulated in micelles to improve its water solubility and plasma bioavailability. For this reason, our observational study is aimed to assess which of these strategies holds the best performance in improving the bioavailability of turmeric extracts when taken as a dietary supplement and examining the impact of turmeric on several biological parameters related to inflammation, such as Erythrocyte Sedimentation Rate (ESR), C-reactive protein (CRP) and Ferritin [39-41], and cardiovascular risks such as cholesterol LDL [42].

## 2 MATERIALS AND METHODS

The observational research was conducted on 30 patients, 15 patients were treated with turmeric (*Curcuma longa L*., rhizome) dried extract titled 95% Curcumin (Curcumin 425 mg/day) conjugated to piperine, and 15 patients were treated with Curcumin (turmeric 286 mg dried extract titled 35%; Curcuminoids 100 mg/day, standardized water-soluble) made in micelles highly absorbed in aqueous solution. All patients are treated with water-soluble Curcumin for recurrent osteo-articular inflammatory diseases (fibromyalgia, multidistrict arthrosis with arthritis). We considered the quantitative variations of laboratory parameters: Erythrocyte Sedimentation Rate (ESR) (men < 15 mm/hr. - 20 mm/hr.), C-reactive protein (CRP) (0.8 mg/L- 3.0 mg/L, until 10 mg/L in some healthy adults), Ferritin (24 to 336 ng/mL for adult males), and cholesterol LDL (<100 mg/dL). Inclusion criteria are reported in Table **1**.

In fasted subjects at least 12 h, we measured serum low-density lipoprotein (LDL) concentration using an automatic analyzer (Advia 1650 Autoanalyzer; Bayer Diagnostics, Leverkusen, Germany). To assess the *in vivo* iron concentrations, serum ferritin levels were measured by performing immunoradiometric assay. For inflammatory biomarkers, blood samples were collected in 3 ml tubes (Vacutainer, Becton Dickinson, UK) with the K_2_EDTA anticoagulant (1.8 mg/ml) under standard conditions and tested within 4 hr of venipuncture, according to ICSH (International Council Standardization in Hematology) recommendations, using the ESR STAT 6 Sed Rate Analyzer (HemaTechnologies). CRP levels were measured by using the immunonephelometry method (Nephelometry, Behring Nephelometer; Dade Behring Marburg GmbH, Germany). High-performance liquid chromatography (HPLC) was performed for the quantification of Curcumin and its metabolites in urine [43]. All analyses were conducted at the same healthcare facility.

## 3 RESULTS

Turmeric-treated patients show a significant difference in the laboratory parameter values selected for this study. The differences are related to the type and consumption of turmeric-dried extract during the time. Patients treated with dried extract titled 95% Curcumin, for 90 days, show a lower value of ESR, CRP, Ferritin, and LDL cholesterol compared with the same laboratory parameters before the introduction of Curcumin into the diet. Also, patients treated with Curcumin report a lower value of ESR, CRP, Ferritin, and LDL cholesterol after the introduction of turmeric dried extract in the diet, but with a major significance compared with those obtained with 95% Curcumin.

As we see in Fig. (**1i**), 95% Curcumin (A), when introduced as supplementation, exerts a beneficial effect by modifying the inflammatory biomarker ESR, with a significant difference before and after 90 days of treatment (*p* < 0.01). The difference in ESR is even more marked in patients treated with Curcuin (B) (*p* <0.001). At baseline (T0), the mean ESR levels for the group treated with 95% Curcumin were 28 mm/hr., whereas the group treated with Curcumin had a slightly higher baseline ESR of 31.33 mm/hr. After 90 days of treatment (T90), both groups exhibited a significant reduction in ESR levels. The 95% Curcumin group showed a decrease to 18.6 mm/hr., while the Curcumin group demonstrated an even more pronounced reduction to 15.6 mm/hr. This result indicates that while both formulations are effective in lowering ESR, the water-soluble Curcumin formulation had a superior anti-inflammatory effect.

Also, the inflammatory index CRP is influenced by the usage of Curcuminoids; Fig. (**1ii**) reports the difference in CRP value, before and after 90 days of treatment with 95% Curcumin (A) and Curcuin (B). It's interesting to note how the difference in CRP values is significant both in 95% of Curcumin treated-patients (*p* <0.001) and Curcuin treated-patients (*p* <0.0001). CRP, in fact, was elevated in both groups at baseline, with the 95% Curcumin group starting at 5.93 mg/L and the Curcuin group at 6.2 mg/L. After 90 days of treatment, both groups experienced substantial reductions in CRP levels, with the 95% Curcumin group decreasing to 2.7 mg/L and the Curcuin group dropping to 2.63 mg/L. The data suggest that both formulations significantly reduce systemic inflammation, with Curcumin again showing slightly greater efficacy in reducing CRP levels.

Ferritin levels, shown in Fig. (**1iii**), which reflect systemic iron stores and inflammation, were also elevated at baseline, with the 95% Curcumin group (A), showing a mean value of 306.14 ng/mL, and the Curcuin group (B), displaying a higher mean of 338.5 ng/mL. After the 90-day treatment period, the 95% Curcumin group showed a modest reduction in ferritin levels to 279.71 ng/mL, while the Curcumin group exhibited a more substantial decrease to 280.0 ng/mL. These findings further highlight the enhanced anti-inflammatory properties of Curcumin in reducing ferritin levels, an important marker for chronic inflammation.

LDL cholesterol was another key biomarker evaluated in the study. As displayed in Fig. (**1iv**), both formulations led to reductions in LDL cholesterol after 90 days of treatment, with Curcuin (B) again demonstrating a more significant effect in lowering cholesterol levels compared to 95% Curcumin (A).

**Fig. (2):**
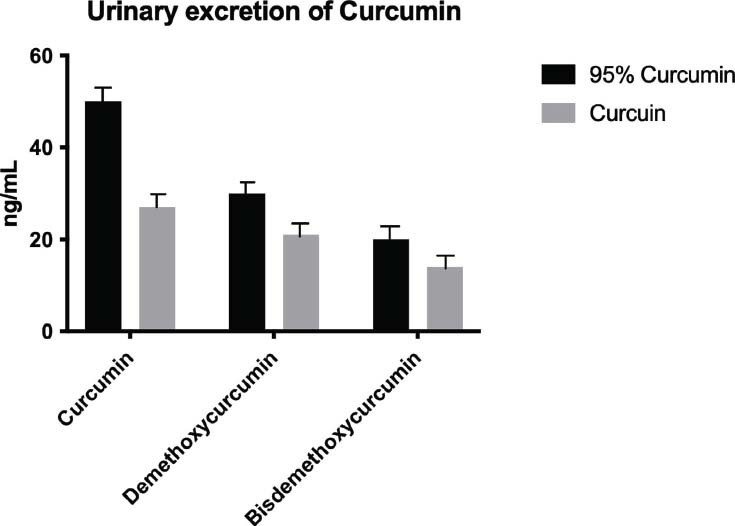
Urinary levels of Curcumin and its metabolites *Demethoxycurcumin* and *Bisdemethoxycurcumin* measured 4 hours after the administration of 95% Curcumin and Curcuin.

To further assess the bioavailability of the two Curcumin formulations, the urinary excretion of Curcumin and its metabolites (Desmethoxycurcumin and *Bisdemethoxycurcumin*) was measured 4 hours after administration (Fig. **2**). Patients treated with 95% Curcumin exhibited higher levels of Curcumin and its metabolites in their urine compared to those treated with curcumin. Specifically, urinary Curcumin concentrations were 50 ng/mL for the 95% Curcumin group and 27 ng/mL for the Curcumin group. Similarly, the excretion of Desmethoxycurcumin was 30 ng/mL in the 95% Curcumin group and 21 ng/mL in the Curcuin group, while *Bisdemethoxycurcumin* levels were 20 ng/mL and 14 ng/mL, respectively.

These findings suggest that the Curcuin formulation, with its improved bioavailability, results in lower urinary excretion, indicating greater systemic absorption and retention compared to the traditional 95% Curcumin formulation. This enhanced absorption likely contributes to the superior anti-inflammatory effects observed with Curcuin in our study.

## 4 DISCUSSION

Curcumin, a polyphenol derived from turmeric, has long been studied for its wide range of pharmacological effects, including anti-inflammatory, antioxidant, and anticancer properties [44]. However, despite its therapeutic potential, Curcumin's clinical application has been hindered by its poor bioavailability, largely due to its limited absorption, rapid metabolism, and quick systemic elimination [45]. To address these issues, various formulations aimed at enhancing Curcumin’s bioavailability have been developed, including those conjugated with piperine and more recently, micelle-based water-soluble formulations such as Curcumin [46, 47].

Our study aimed to compare the systemic anti-inflammatory effects of a traditional fat-soluble Curcumin formulation (95% Curcumin with piperine) and an innovative water-soluble Curcumin formulation (Curcuin) to evaluate the efficacy of these compounds in modulating key inflammation-related biomarkers: erythrocyte sedimentation rate (ESR), C-reactive protein (CRP) and ferritin, and their effect on LDL cholesterol. Both formulations demonstrated significant improvements in the selected biomarkers; however, the Curcumin formulation showed a greater and more statistically significant effect on reducing ESR, CRP, and ferritin levels, as well as LDL cholesterol.

The observed differences between the two formulations can be attributed to their distinct bioavailability profiles. Previous studies have shown that the combination of Curcumin with piperine enhances absorption by inhibiting hepatic and intestinal glucuronidation, leading to an increase in Curcumin bioavailability [38, 48]. However, despite this improvement, the bioavailability of Curcumin remains suboptimal, as much of the compound is still rapidly metabolized and excreted. In contrast, the micelle-based formulation used in Curcumin has been shown to significantly improve Curcumin’s solubility and plasma bioavailability, enabling more efficient delivery to target tissues.

The improved bioavailability of Curcumin is further supported by the results of the urinary excretion analysis performed in our study. Patients treated with Curcumin exhibited lower levels of Curcumin and its metabolites in their urine compared to those treated with 95% Curcumin, suggesting that a higher proportion of the compound was absorbed and utilized in the body. This finding is consistent with previous reports that micellar formulations can enhance the systemic retention of Curcumin, thereby improving its therapeutic efficacy.

Inflammation is a key driver of numerous chronic diseases, including arthritis, cardiovascular disease, and metabolic disorders. The significant reductions in ESR and CRP observed in both treatment groups highlight Curcumin’s potential as a therapeutic agent in the management of systemic inflammation. However, the superior efficacy of Curcumin suggests that improving the bioavailability of Curcumin may be critical for achieving its full therapeutic potential. Moreover, the reduction in LDL cholesterol levels in patients treated with Curcuin indicates that Curcumin may also exert beneficial effects on lipid metabolism, further supporting its use as an adjunctive therapy for managing both inflammation and associated metabolic conditions.

Despite these promising results, there are several limitations to our study that warrant further investigation. First, the relatively small sample size and short duration of treatment limit the generalizability of our findings. While the observed reductions in inflammation and cholesterol are encouraging, larger-scale studies with longer follow-up periods are needed to confirm these effects and evaluate the long-term safety and efficacy of Curcumin supplementation. Furthermore, while our study focused on ESR, CRP, ferritin, and LDL cholesterol as markers of inflammation and metabolic health, future studies should consider a broader range of biomarkers, including pro-inflammatory cytokines (*e.g.,* TNF-α, IL-6), oxidative stress markers, and additional lipid parameters (*e.g.,* HDL cholesterol, triglycerides), to gain a more comprehensive understanding of Curcumin’s effects.

Another important aspect to explore is the dose-response relationship for both 95% Curcumin and Curcumin. Although Curcuin showed greater efficacy at a lower dose (100 mg/day) compared to 95% Curcumin (425 mg/day), further studies are needed to determine the optimal dosing regimen for each formulation. This is particularly important given the increasing interest in Curcumin as a natural alternative to conventional anti-inflammatory drugs, which are often associated with adverse side effects.

In addition to exploring optimal dosing, future research should also focus on the pharmacokinetics and pharmacodynamics of Curcumin in different patient populations. Factors such as age, gender, genetic polymorphisms, and underlying health conditions can influence Curcumin metabolism and therapeutic response, and personalized approaches may be required to maximize its benefits.

In conclusion, our findings demonstrate that both Curcumin formulations exhibit significant anti-inflammatory and cholesterol-lowering effects, with Curcumin showing superior efficacy likely due to its enhanced bioavailability. While these results are promising, they also underscore the need for more extensive clinical studies to fully elucidate the therapeutic potential of Curcumin, optimize dosing strategies, and explore its broader applications in chronic inflammatory and metabolic diseases. Given the growing burden of these conditions worldwide, Curcumin, especially in its more bioavailable forms, may offer a valuable addition to the current armamentarium of therapeutic interventions.

## CONCLUSION

As we had hypothesized, both turmeric-dried extracts have exerted an anti-inflammatory role, reducing ESR, CRP, Ferritin, and cholesterol LDL values. Herein, we have observed how 100 mg of Curcumin has a similar and more powerful action than 425 mg of Curcumin-piperine. So, Curcumin can be used as a well-tolerated dietary addition to conventional medications. But, additional investigation is needed to resolve doubts regarding Curcumin dosage form, dose, and medication frequency. However, the increased bioavailability of Curcumin shortly will likely bring this promising natural product to the forefront of therapeutic agents for the treatment of multiple chronic diseases that require considerable healthcare resources.

## Figures and Tables

**Fig. (1):**
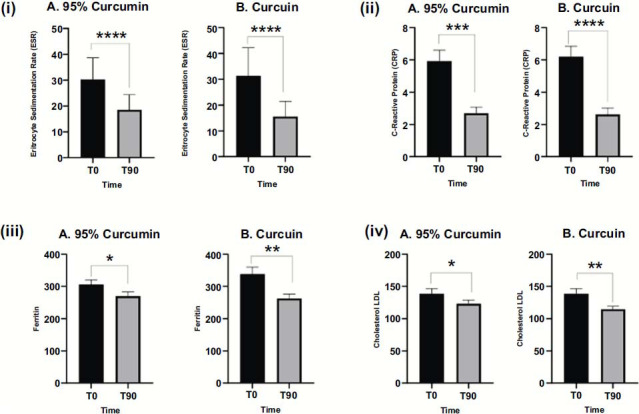
A two-tailed unpaired *t*-test was performed on normalized values from both treated groups. Significance is attributed in the following way: ns for *p*-values above 0.05; * for *p*-values between 0.05 and 0.01; ** for *p*-values between 0.01 and 0.001; *** for *p*-values between 0.001 and 0.0001; and **** for *p*-values smaller than 0.0001. (**i**) ESR blood level in patients treated with 95% Curcumin (**A**) and Curcuin (**B**) before and after 90 days of turmeric dried extract administration. Both turmeric dried- extracts are able to lower ESR. (**ii**) CRP serum level in patients treated with 95% Curcumin (**A**) and Curcuin (**B**) before and after 90 days of turmeric dried extract administration. Both turmeric dried- extracts are able to lower CRP levels. (**iii**) Ferritin serum level in patients treated with 95% Curcumin (**A**) and Curcuin (**B**) before and after 90 days of turmeric dried extract administration. Curcuin significantly influences ferritin serum level in contrast to 95% Curcumin. (**iv**) LDL serum level in patients treated with 95% Curcumin (**A**) and Curcuin (**B**) before and after 90 days of turmeric dried extract administration. A two-tailed unpaired *t*-test was performed on normalized values from both treated groups Curcuin mildly influences cholesterol LDL serum levels.

**Table 1:** Cohort inclusion criteria.

**Inclusion Criteria**	**Value**
Sex	Male
Age	35-55
Drugs, NSAIDS, Steroids	None
Comorbidities	None
Erythrocyte Sedimentation Rate (ESR)	>20
C-reactive Protein (CRP)	>3
Low Density lipoprotein (LDL) Cholesterol	>130
Ferritin	<250

## Data Availability

All data generated or analyzed during this study are included in this published article.

## LIST OF ABBREVIATIONS

COX-II: Cyclooxygenase II
CRP: C-Reactive Protein
DNMT: DNA Methyltransferase
ESR: Erythrocyte Sedimentation Rate
GSH: Glutathione
LDL: Low-Density Lipoprotein
RNS: Reactive Nitrogen Species
ROS: Reactive Oxygen Species
SOD: Superoxide Dismutase
TNF-α: Tumor Necrosis Factor-α

## STANDARDS OF REPORTING

STROBE guidelines were followed.
